# How Does a Walker Pass Between Two People Standing in Different Configurations? Influence of Personal Space on Aperture Passing Methods

**DOI:** 10.3389/fpsyg.2019.02651

**Published:** 2019-12-04

**Authors:** Takayuki Tomono, Ryosaku Makino, Nobuhiro Furuyama, Hiroyuki Mishima

**Affiliations:** ^1^Graduate School of Human Sciences, Waseda University, Saitama, Japan; ^2^Faculty of Human Sciences, Waseda University, Saitama, Japan

**Keywords:** aperture passability, aperture passing, personal space, shoulder rotation angle, shoulder rotation strategy, trajectory strategy

## Abstract

Most studies on aperture passability focus on aperture passing involving non-human physical objects. In this study, we examined experimentally how participants pass between two box-shaped frames and between the same frames, each with a human confederate in it, facing various directions. Seven configuration conditions were set up, six of which differed in terms of the human confederates’ sets of directions in the two frames: face-to-face, back-to-back, facing toward or away from the participants, facing leftward or rightward from the participants’ perspective, and the empty frames condition without human confederates. There were seven aperture-width conditions—50, 55, 60, 65, 70, 75, and 80 cm—and participants walked at their normal speed through the apertures. We found that the participants’ shoulder rotation angle in the face-to-face condition was significantly greater than that in the empty frames condition. Further, the participants preferred to rotate their shoulders counterclockwise when our confederates in the aperture faced leftward, and clockwise, when they faced rightward. These results suggest that people change their passing-through methods by considering the social nature of the aperture as well as its width.

## Introduction

Our living environment comprises of many types of furniture and apertures, and we need to safely pass through apertures constructed by the furniture. For instance, we have to go through the crowds safely in crowded places and move through the desks in the classroom. How can we safely pass through such apertures in the living environment?

Most studies on aperture passability (Warren and Whang, 1987; Flascher, 1998; Hackney and Cinelli, 2011; Higuchi et al., 2011; Muroi et al., 2017) focus on aperture passing involving non-human physical objects such as doorways and aisles, and the emphasis seemingly tends to be on safety. Many everyday activities, however, are conducted in the presence of other people. Walking through a crowd to go to a destination is no exception, and it should be done socially adaptively as well as physically safely.

When introducing the notion of *field of safe travel*, which later developed as the idea of affordances, Gibson and Crooks (1938) discussed it not only in physical terms, but also in terms of social interaction. The field of safe travel is defined as follows:

Within the boundaries of the road lies, according to our hypothesis, an indefinitely bounded field, which we will name the field of safe travel. It consists, at any given moment, of the field of possible paths that the car may take unimpeded (*ibid.*: p. 454).

In the same paper, it was pointed out that “the driver does take into account the field of the other person” (*ibid.*: p. 467). That is, the drivers see each other and adjust their field of safe travel such that they do not overlap or collide with each other. The present study investigated how people pass by two humans in terms of social aspects of affordances as advocated by Gibson and Crooks (1938) 80 years ago. We consider this as one of the important questions to address in the human movement science.

### Previous Studies on Aperture Passing

Warren and Whang (1987) showed that people rotate their shoulder when the aperture to pass through is approximately or less than 1.30 times their shoulder width. This result thus suggests that the critical ratio of the aperture width to the person’s shoulder width (i.e., pi-number), obtained by dividing the “passable” and the “impassable” judgments for actual aperture passing is 1.30 (*ibid.*).

Aperture passability changes depending on the background of the passer (i.e., age, experience in sports, etc.), and situations (i.e., running vs. walking, etc.). For example, Hackney and Cinelli (2011) identified that when the elderly walked though apertures, the critical pi-number for shoulder rotation was 1.60. This is much larger than the pi-number reported for younger adults (i.e., undergraduates) in the Warren and Whang (1987) study, which is 1.30, mentioned above. Another example comes from the study by Higuchi et al. (2011), which demonstrated that athletic experience has an impact on the critical pi-number for shoulder rotation in the task of running through apertures. When American football players were asked to run though apertures (composed of two balloons with pictures of human defenders on them), their shoulder rotation angle was smaller than that of tennis players, who were not typically engaged in running through narrow apertures. However, no difference was found between American football players and the other sport players, when they were asked to walk through apertures. Furthermore, the nature of the environment may affect the passability of apertures. For example, Flascher (1998) found that people needed more margins of safety when they walked through a wooden doorway with studded nails than when they walked through apertures without them.

People also change the method for passing through an aperture adaptively to their body condition. For example, Muroi et al. (2017) demonstrated that when hemiplegic patients walked through apertures, they were less likely to bump into obstacles, by which the aperture is formed, as they rotated their shoulder such that the paretic side entered the aperture earlier than the other side.

What has not been addressed fully in the literature is the following question: Does aperture passability and how one passes through it change depending on whether one passes by humans or non-human physical objects? Hackney et al. (2015) compared how people pass an aperture between non-human objects (two poles) and that between humans (two female confederates for the experiment) who stood facing the participant. It turned out that the critical pi-number (aperture width/shoulder width) for walking by humans was 1.70, which was much greater than that for passing by non-human objects, i.e., 1.30 (Hackney et al., 2015). The authors suggested that these results were because the participants may have accounted for the *personal space* of the confederates.

However, Hackney et al.’s (2015) study has at least two limitations; one pertaining to personal space and the other related to experimental control. Specifically, Hackney et al.’s (2015) study, as well as most other studies, did not examine the anisotropic structure of personal space. Personal space is the invisible but perceivable space surrounding a person, and maintains or changes its shape, depending on the situation (Sommer, 1959; Hall, 1966; Tanaka, 1973; Roger and Schalekamp, 1976). Studies in social psychology have demonstrated that personal space is “anisotropic” in that its shape is like an egg from the top view, with the front being relatively pointed and elongated, and the back round (Tanaka, 1973; Gérin-Lajoie et al., 2005). Tanaka (1973) conducted an experiment in which the participant was asked to approach the standing confederate from eight directions, and to stop walking when he felt uncomfortable. The result showed that personal distance was larger in the following order: in front of the confederate > at an angle of ±45° diagonally in front of the confederate > right from the sides of the confederate > at an angle of ±135° diagonally behind the confederate > right behind the confederate (Tanaka, 1973), with the front being defined as at an angle of zero degree.

**FIGURE 1:**
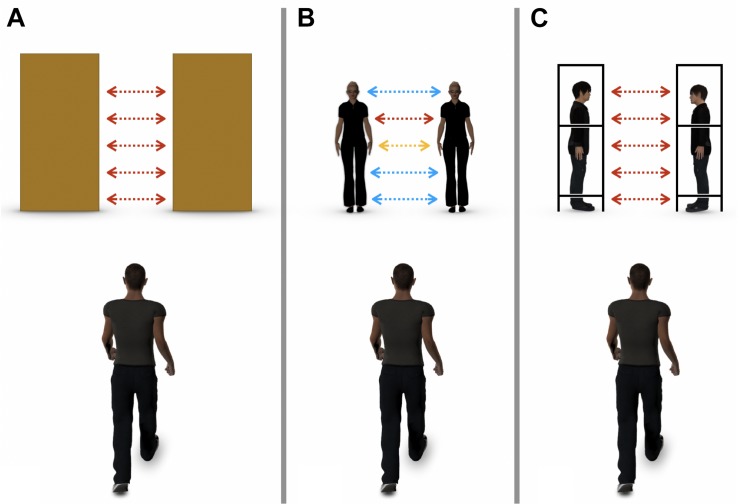
Comparison of experimental conditions in studies on aperture passability. **(A)** Warren and Whang (1987); the participant passed between two objects, the distance between which was the same regardless of the level of obstacles. **(B)** Hackney et al. (2015); the participant passed between two persons, the distance between whom was varied depending on the level of human body (eye-level, shoulder-level, etc.). **(C)** The current study; the participant passed between two box-shaped frames, each with a human confederate. The distance between the two frames was controlled to be the same regardless of the level.

The other limitation of Hackney et al.’s (2015) study is that it is not yet clear whether the participants changed their passing methods (shoulder rotation angle, walking velocity, etc.) owing to the different geometrical shapes of the aperture in the object-obstacle and human-obstacle conditions (Figure 1). As shown in Figure 1, in Warren and Whang’s (1987) study, each participant passed between two objects, the distance between which was the same regardless of the level of obstacles (Figure 1A). In contrast, in Hackney et al.’s (2015) study, participants passed between two persons, the distance between whom varied depending on the level of the human body (eye-level, shoulder-level, etc.); thus, the reason for the change in passing methods cannot be determined (Figure 1B).

### Research Question

**FIGURE 2:**
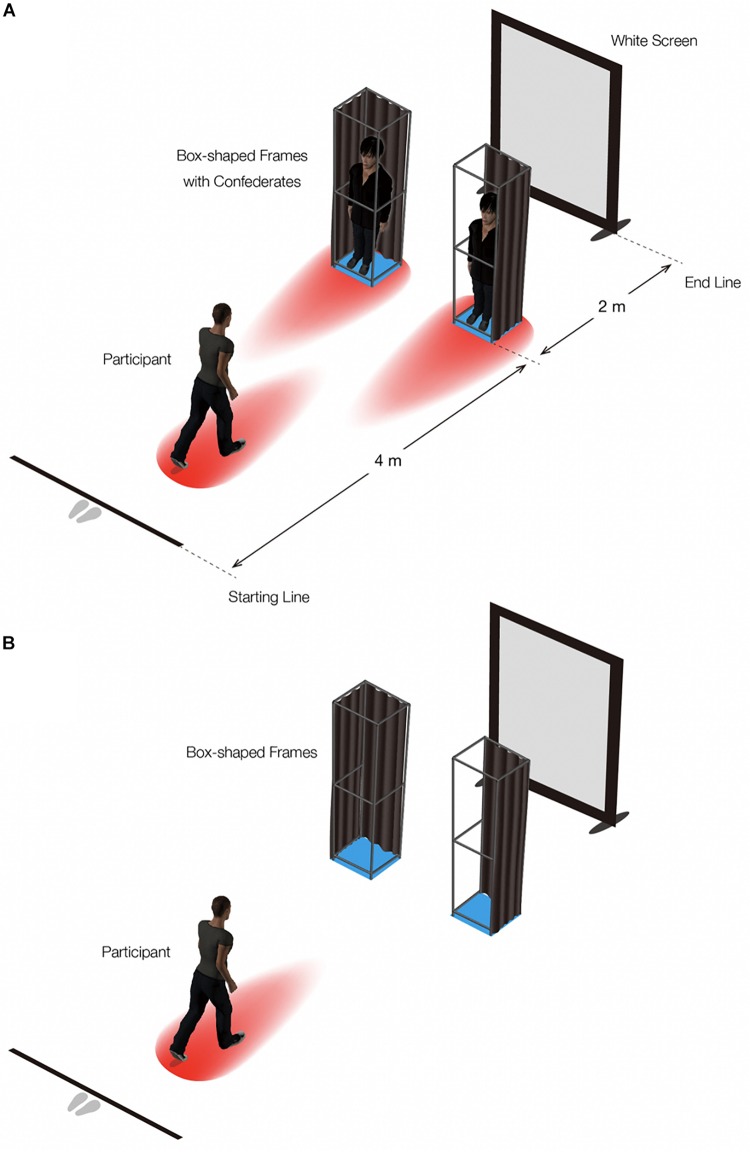
Experimental setting. The blue squares and the space above them each represent the physical space a box frame occupies; the red egg-like shapes and the space above them each represent an anisotropic personal space of each person. In **(A)**, the confederates in the box frames both face toward the participant. In **(B)**, the box frames are empty.

In the present study, to solve these problems, we attempted to observe the passing methods used in walking through an aperture composed of two humans. First, we looked at how people actually pass between humans in differently configured conditions (to be described below), using the trajectory and velocity of locomotion, timing, extent and direction of shoulder rotation, etc., as indices of how and when the passability judgment changes in different conditions. Second, we ensured that the geometrical shape of the apertures was constant, using two box-shaped frames with and without the presence of a human confederate in it, facing various directions (Figures 1C, 2). By doing so, we examined the impact of the presence of humans (and their personal space) in different configurations on aperture passability judgment without introducing the aforementioned confounding factor.

We thus address the following more specific questions: How do people pass through apertures between two humans? Do they make changes in terms of when to start rotating their shoulder, the extent and direction of shoulder rotation, the route to take, and how fast to move before and during walking through an aperture between two humans? Are these changes made according to what constitutes the aperture (box-shaped frames vs. frames with a human in it) and the configurations of the humans inside the frames, if the shape of the frames is kept constant?

### Hypotheses

Our hypotheses, which are based on anisotropic personal space, are as follows: Participants will rotate their shoulder to a greater extent and slow down before and during passing between two humans facing each other than in other configurations. This is because, if personal space is an anisotropic shape, the aperture might in effect be perceived as narrower in the former case for the same physical aperture width; humans have more personal space in the front than in the back or on the sides, which the passer would presumably not want to invade. Participants will rotate their shoulder earlier during passing between two humans when those humans are them. This is because two standing humans’ personal space would extend in front of them and participants would invade their personal space earlier than in other cases. In the humans both facing leftward or rightward from the participant’s perspective, the participants would change the trajectory of walking to avoid passing near the two standing humans’ front side because personal space is deeper on the front side. Now, when an aperture between two humans is narrow in the side-way condition, the participants would have to rotate their shoulder.

## Experiment

To test the hypotheses presented above, we measured shoulder rotation angles, direction of shoulder rotation, distance for shoulder rotation onset, walking trajectories, and velocities of participants who passed through an aperture between two humans (confederates) standing facing various directions. Note that in this experiment, the confederates who formed an aperture each entered an identical box-shaped frame to keep the inside contours of the apertures straight and parallel with each other, despite the “bumpy” shape of their own body. The experimental procedures were approved by the Academic Research Ethical Review Committee at Waseda University.

### Methods

#### Participants

The sample comprised ten male participants (mean height: 170.9 ± 5.2 cm; mean shoulder width: 39.2 ± 2.0 cm; mean age: 20.8 ± 3.0 years). The participants’ shoulder widths were controlled to be the same (±2 cm). The experimenter recorded the height, shoulder width, and age at the end of the experiment. All participants had normal or corrected-to-normal vision. None reported a neurological or musculoskeletal disorder or recent injury. Participants were compensated for their participation.

#### Apparatus

The experiment was conducted in a room (height: 3.0 m; width: 3.8 m; depth: 9.8 m) with a carpet on the floor. The distance from the starting position to the end position was 6.0 m. At 4.0 m from the starting position was an aperture formed by two box-shaped frames (height: 210.0 cm; width: 45.0 cm, depth: 30.0 cm), which were made of plastic pipes (3.8 cm outer radius). Two male confederates, wearing black clothes, entered and stood at the center of the box-shaped frame (the height was 173.0 cm and the shoulder width was 41.5 cm for the left confederate; the height was 174.0 cm, and the shoulder width 41.5 cm for the left confederate), and they were instructed not to move their head and body during a trial^1^. The back and outer side of each box frame from the participant’s viewpoint was covered with brown curtains (Figure 2). A white screen (height: 2.2 m; width: 1.6 m) was placed as a backdrop on the wall behind the aperture to keep the background surface the same throughout the experiment as much as possible (Figure 2).

All data (shoulder rotation angles during walking, preferred shoulder rotation direction, walking trajectories, and walking velocities) were measured with a three-dimensional optical motion tracking system (Optitrack, NaturalPoint, Inc., United States) at a sampling frequency of 100 Hz. A total of 17 passive infrared retroreflective (hereafter, IRR for short) markers were employed: four markers were on the participant’s head, one on the top, one on each side, and one on the back of the head. Three markers were on the participant’s shoulders and neck: one on each acromion and one on the root of the neck (C7). One marker was on each ankle. In this paper, we only report on the data obtained from the two IRR markers on the shoulders.

#### Procedure

We used a within-participant design for the experiment, and all participants performed the task in all conditions. The participants were instructed to walk at their normal speed through an aperture between two humans in various configurations in box-shaped frames or between empty box-shaped frames of the same specification without human confederates in them.

**FIGURE 3:**
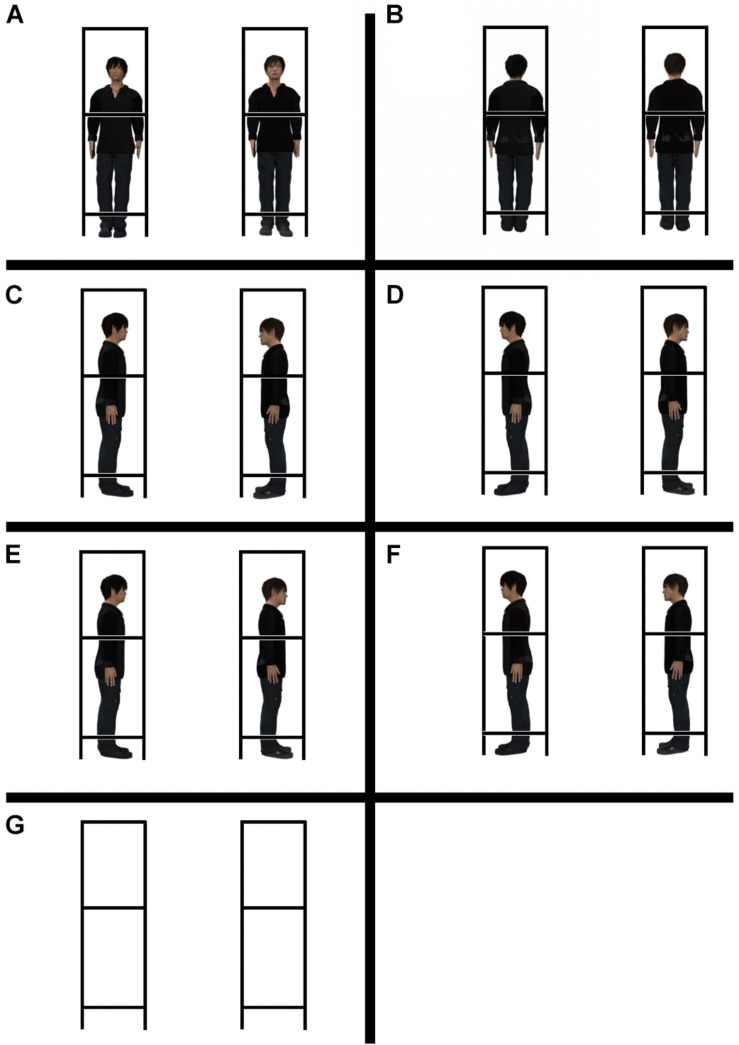
Seven configuration conditions. **(A)** facing condition, **(B)** away condition, **(C)** face-to-face condition, **(D)** back-to-back condition. **(E)** Rightward condition, **(F)** leftward condition, **(G)** empty-frame condition.

The experiment had two factors. One factor was a configuration factor, and the other was an aperture-width factor. The seven configuration conditions were set up in terms of the configuration of human confederates: the facing condition (confederates aligned side by side facing the participants), away condition (confederates aligned side by side facing away from the participants), rightward condition (confederates aligned sideways, facing rightward), leftward condition (confederates aligned sideways, facing leftward), face-to-face condition (confederates facing each other), back-to-back condition (confederates aligned with their backs facing each other), and empty-frame condition (empty box-shaped frames) (Figure 3). The aperture widths^2^ were presented in the range 50.0–80.0 cm in 5-cm increments or decrements, constituting seven aperture-width conditions.

The experiment was conducted in two blocks (Table 1). In each block, the aperture-width conditions were presented such that an increment sequence and a decrement sequence alternated. Fifty percent of the participants experienced the increment sequence first, and the remaining participants the decrement sequence first. The configuration conditions were randomly assigned for the sequences in the first block. In the second block, the configuration conditions were presented in the same order as the first block, but for counter-balance, the sequence in which aperture-width conditions were presented was reversed.

**TABLE 1:** An example of the experimental sequence.

	**Trial 1**	**Trial 2**	**Trial 3**	**Trial 4**	**Trial 5**	**Trial 6**	**Trial 7**
Block 1	C_4_ × a	C_3_ × d	C_7_ × a	C_2_ × d	C_6_ × a	C_5_ × d	C_1_ × a
Block 2	C_4_ × d	C_3_ × a	C_7_ × d	C_2_ × a	C_6_ × d	C_5_ × a	C_1_ × d

*a, ascending sequence of the aperture-width conditions. d, descending sequence of the aperture-width conditions. C_*x*_, seven configuration conditions (randomly assigned to each participant).*

The participants initially stood upright at the starting position while facing away from the aperture. After receiving a prompt from the experimenter, the participants turned around and started walking toward and through the aperture, until reaching the goal position. When the participants finished the task, they were instructed to turn to the right or left in a random manner and return to the starting position. We did this to avoid introducing a bias to the data; otherwise, the participants may turn only to one side to return to the starting position, and they may tend to turn their shoulders to only the one side or walk to one side of the aperture. There were 98 trials in total (7 configuration conditions × 7 aperture-width conditions × 2 order conditions, i.e., the aperture width increases or decreases as the experiment proceeds). The aperture width was changed by the confederates while the participants returned to the starting position. The participants were not allowed to see the aperture while it was being changed.

### Data Analysis

#### Shoulder Rotation Angles

**FIGURE 4:**
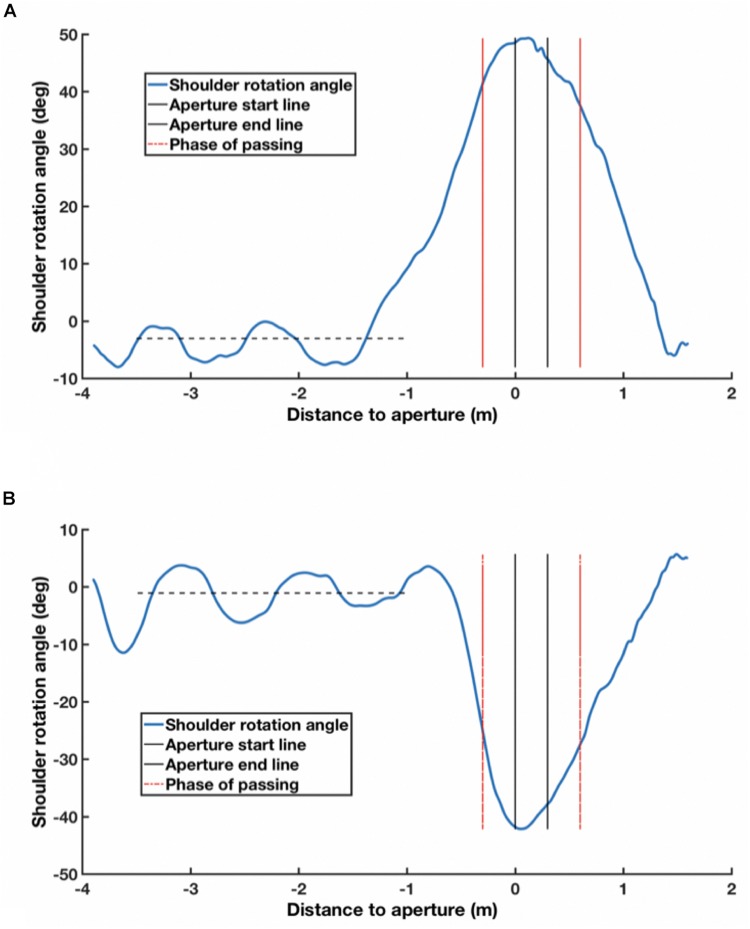
Shoulder angle during one trial. **(A)** Shoulder angle in counterclockwise shoulder rotation. **(B)** Shoulder angle in clockwise shoulder rotation. **(A,B)** Show examples of, respectively, counterclockwise and clockwise rotations of the shoulder. The sign of the shoulder angle in **(A,B)** is positive and negative, respectively. The black dotted line represents the average shoulder rotation angle in the approaching phase (from 3.5 to 1.0 m before the aperture).

Following Hackney et al. (2015), we analyzed the two IRR markers on the participants’ shoulders. Each time series was smoothed by a sixth-order Butterworth filter with a low-pass cut-off frequency of 15 Hz, using MATLAB (MathWorks, version 9.1). The geometric center of the body for each participant on the horizontal plane was calculated by averaging the positions of the two IRR markers. The shoulder angles were derived from the inclination of a line joining the two IRR markers to the frontal parallel plane of the box-shaped frames constituting the apertures. The maximum or minimum shoulder angle was determined for the *phase of passing* (POP), i.e., the range between a point 0.3 m before the front side of the frame and that 0.3 m after its backside. If the maximum or minimum shoulder angle for the POP fell outside three standard deviations of the average shoulder angles during approaching (defined as steady-state locomotion from 3.5 to 1.0 m before the apertures), the shoulder was considered to have rotated, and otherwise it was not (Figure 4).

#### Direction of Shoulder Rotation

The direction of shoulder rotation (if it did indeed take place as defined above) was determined by the plus or minus sign associated with the shoulder angle. If the sign of the shoulder angle was positive, a counterclockwise shoulder rotation was considered to take place. Otherwise, a clockwise shoulder rotation was considered to have occurred (Figure 4).

#### Distance for Shoulder Rotation Onset

The distance to the aperture from the center of a participant’s body when the shoulder rotation started was measured. The criterion for the onset of rotation was the same as stated above; when the shoulder rotation angle fell outside three standard deviations of the average shoulder angles during approaching, the shoulder was considered to have significantly rotated (Figure 4).

#### Deviation of the Medial-Lateral Position (MLP) From the Center Line Between the Box-Shaped Frames

**FIGURE 5:**
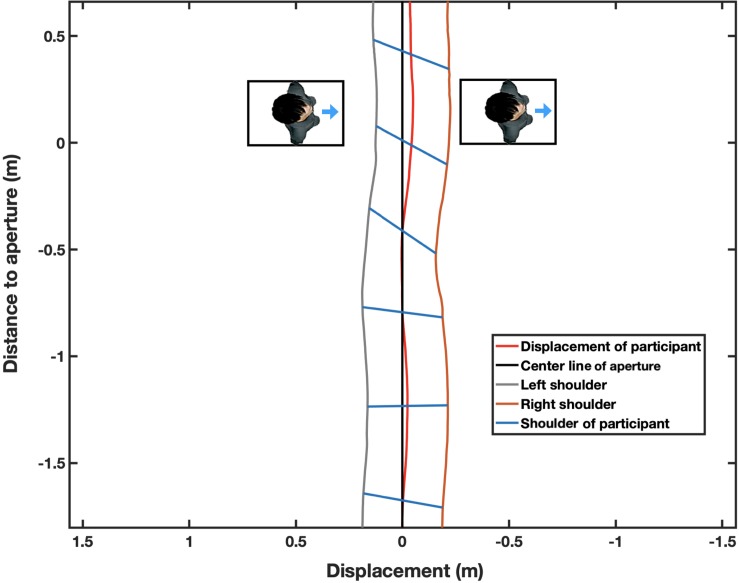
Bird’s eye view of the experimental setting showing how a participant performed one of the tasks in the 55 cm aperture-width condition. This figure is an enlarged view from a distance of -1.5 to 0.5 m to the aperture. The quasi-horizontal blue lines represent the traces of the participant’s shoulder, the red line indicates divergence off the center line in a trial in the rightward condition. It can be seen that the shoulder is rotated clockwise to avoid contacting obstacles, including rightward facing confederates, and the Medial-Lateral Position from the center line is diverged to the right. The time interval of shoulder traces (blue line) was 0.3 s.

We wanted to determine whether the two standing persons had any impact on the trajectory of the participants’ walking. Specifically, we wanted to observe whether the walkers would diverge either to the right or to the left off the center line of the aperture according to the two standing persons’ anisotropic personal space. For each participant, the geometric center of the body on the horizontal plane was calculated by averaging the positions of the two IRR markers. The lateral divergence of the body from the center line was estimated by determining the distance between the central line of the aperture (which was not printed on the floor) and the geometric center of the participant from the start to end of the aperture (Figure 5). If the estimated divergence had a minus sign, it meant that the participant tended to diverge to the right and vice versa.

#### Velocity in the Approaching Phase and Passing Phase

The velocities of walking during a trial on the horizontal plane were calculated by differentiating the horizontal displacement of the geometric center of the participant’s body with reference to time. The approaching velocity was defined as the average velocity from 3.5 to 1.0 m before reaching the apertures. The passing velocity was defined as the average velocity from the start of the aperture to its end.

## Results

### Shoulder Rotation and Its Angle

The data obtained for the ten participants were analyzed. The participants performed aperture crossing under the same set of conditions, two times: once with increase and once with decrease in the aperture width. Thus, in this analysis, the average of two shoulder angles in the POP was used as the representative value. The mean values of the shoulder angle and 95% confidence interval (CI) in each condition and each aperture width have been presented in Table 2.

**TABLE 2:** Mean magnitude of shoulder angle (deg).

**Configuration conditions**	**Aperture width**						
	**50 cm**	**55 cm**	**60 cm**	**65 cm**	**70 cm**	**75 cm**	**80 cm**
Face-to-face	42.12 [38.16, 46.08]	31.71 [27.25, 36.17]	22.29 [17.47, 27.10]	11.56 [9.25,13.86]	7.61 [4.78,10.44]	7.31 [4.27,10.36]	7.58 [4.83,10.34]
Facing	44.73 [40.99, 48.47]	31.32 [26.20, 36.43]	21.00 [16.58, 25.41]	10.46 [8.11,12.80]	7.58 [4.76,10.41]	6.73 [4.11,9.35]	6.14 [3.55, 8.73]
Rightward	42.29 [35.65, 48.94]	28.74 [23.60, 33.89]	20.44 [14.98, 25.89]	13.75 [10.06,17.44]	10.32 [7.18,13.46]	9.12 [6.46,11.78]	7.66 [5.29,10.04]
Leftward	41.95 [36.34, 47.55]	29.54 [25.17, 33.91]	16.02 [13.70,18.33]	10.97 [8.64,13.30]	8.79 [6.09,11.48]	6.61 [3.66, 9.57]	7.10 [3.94,10.26]
Back-to-back	42.85 [37.62, 48.07]	28.11 [22.83, 33.39]	18.31 [15.17, 21.44]	9.51 [7.33,11.70]	7.34 [4.56,10.12]	7.12 [4.25, 9.99]	6.87 [3.98, 9.76]
Away	38.25 [32.58, 43.92]	24.96 [19.38, 30.54]	15.20 [11.63,18.78]	9.87 [6.71,13.03]	7.98 [5.14,10.82]	6.94 [4.26, 9.62]	7.05 [4.36, 9.75]
Empty-frame	36.72 [29.12, 44.32]	21.86 [17.44, 26.28]	15.25 [11.19,19.32]	8.74 [6.15,11.32]	7.25 [4.46,10.03]	6.71 [3.71,9.72]	6.36 [3.29, 9.43]

*Values in square brackets indicate 95% confidence intervals.*

**FIGURE 6:**
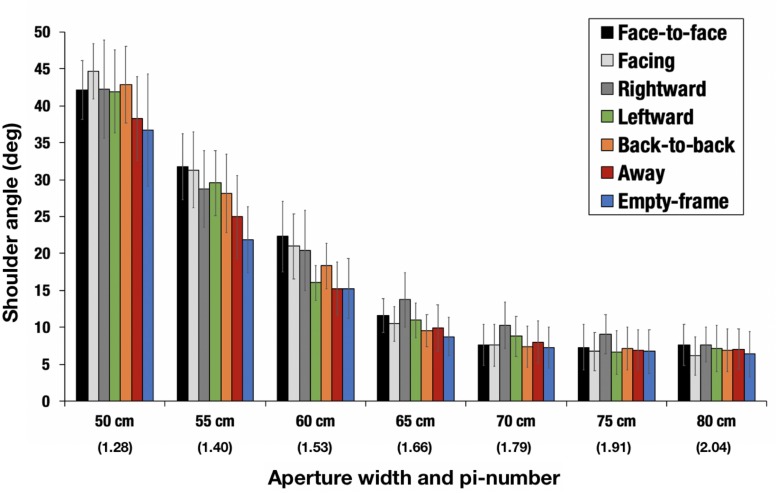
Magnitude of shoulder angle. The parenthetical number indicates pi-number, i.e., aperture width divided by the mean shoulder width of the participants. The error bars indicate 95% CI. The magnitude of the shoulder angle in the face-to-face condition was significantly larger than that in the empty-frame condition (*adjusted p* < 0.05). The magnitude of the shoulder angle became larger as the aperture-widths decreased; specifically, 50 cm (π = 1.28) > 55 cm (π = 1.40) (*adjusted p* = 0.0004) > 60 cm (π = 1.53) (*adjusted p* = 0.0011) > 65 cm (π = 1.66) (*adjusted p* = 0.0452) > 70 cm (π = 1.79) (*adjusted p* = 0.0452).

A two-way repeated measures analysis of variance (7 configurations × 7 aperture-widths) was conducted for the representative values of shoulder angles, as defined above. Significant main effects of configurations [*F*(6,54) = 3.2758, *p* = 0.0080, $\eta_{G}^{2}$ = 0.0241, *power* = 0.9998] and aperture-widths [*F*(6,54) = 44.3911, *p* < 0.001, $\eta_{G}^{2}$ = 0.6387, *power* = 1.0000] were identified (Figure 6). No significant interactions [*F*(36,324) = 1.1827, *p* = 0.2250, $\eta_{G}^{2}$ = 0.0245, *power* = 0.4207] were confirmed. The Holm–Bonferroni method, also known as the sequentially rejective Bonferroni test, was applied to the configurations, and it was revealed that the magnitude of the shoulder angle in the face-to-face condition was significantly larger than that in the empty-frame condition (*adjusted p* = 0.0492). In addition, the same test applied to the aperture-widths revealed that the magnitude of the shoulder angle becomes larger as the aperture-widths become narrower, specifically, 50 cm (π = 1.28) > 55 cm (π = 1.40) (*adjusted p* = 0.0004), >60 cm (π = 1.53) (*adjusted p* = 0.0011), >65 cm (π = 1.66) (*adjusted p* = 0.0452), and >70 cm (π = 1.79) (*adjusted p* = 0.0452). The significant shoulder rotation disappeared when the aperture was set at 70 cm (π = 1.79) or larger.

### Preferred Direction of Shoulder Rotation

**FIGURE 7:**
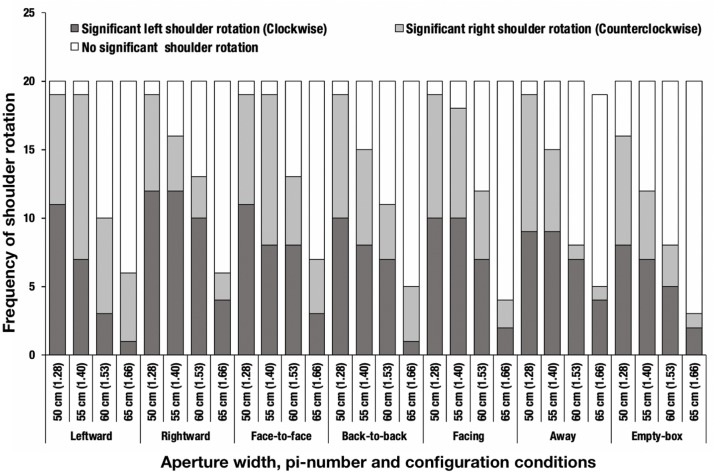
Frequency of shoulder rotation. The parenthetical number indicates pi-number, i.e., aperture width divided by the mean shoulder width of the participants. One data point from the away condition was excluded as an outlier, as described in the main text.

Next, we investigated if the participants exhibited a preference to any direction in which they rotated their shoulder. We compared the frequency of shoulder rotation by direction, using the data for the aperture of 65 cm (π = 1.66) or less, because shoulder rotation seldom occurred in conditions pertaining to the aperture of 70 cm (π = 1.79) or greater. The frequencies of shoulder rotation by direction for the aperture of 65 cm (π = 1.66) or less are listed in Table 3. We excluded one of the data from the “away” condition as an outlier, because the participant turned his shoulder both in the right and left directions in one trial, such that the shoulder angles in both cases exceeded 3 SDs of the average rotation angle in POP. This outlier explains the occurrence of an indentation in the away condition and the aperture-width of 65 (π = 1.66) cm in Figure 7.

**TABLE 3:** Frequency of shoulder rotation by direction of rotation and result of residual analysis (*p* < 0.05) of Chi-squared test.

		**Significant shoulder rotation**								
**Configuration**	**Aperture width**									
**conditions**	**and pi-number**	**Clockwise**			**Counterclockwise**			**None**		
Leftward	50 cm (1.28)	11			8			1	▼	(*p* = 0.0081)
	55 cm (1.40)	7			12	△	(*p* = 0.0081)	1	▼	(*p* = 0.0081)
	60 cm (1.53)	3			7			10		
	65 cm (1.66)	1	▼	(*p* = 0.0102)	5			14	△	(*p* = 0.0081)
Rightward	50 cm (1.28)	12	△	(*p* = 0.0290)	7			1	▼	(*p* = 0.0081)
	55 cm (1.40)	12	△	(*p* = 0.0290)	4			4		
	60 cm (1.53)	10			3			7		
	65 cm (1.66)	4			2			14	△	(*p* = 0.0081)
Face-to-face	50 cm (1.28)	11			8			1	▼	(*p* = 0.0081)
	55 cm (1.40)	8			11	△	(*p* = 0.0162)	1	▼	(*p* = 0.0081)
	60 cm (1.53)	8			5			7		
	65 cm (1.66)	3			4			13	△	(*p* = 0.0162)
Back-to-back	50 cm (1.28)	10			9			1	▼	(*p* = 0.0081)
	55 cm (1.40)	8			7			5		
	60 cm (1.53)	7			4			9		
	65 cm (1.66)	1	▼	(*p* = 0.0102)	4			15	△	(*p* = 0.0038)
Facing	50 cm (1.28)	10			9			1	▼	(*p* = 0.0081)
	55 cm (1.40)	10			8			2	▼	(*p* = 0.0256)
	60 cm (1.53)	7			5			8		
	65 cm (1.66)	2	▼	(*p* = 0.0290)	2			16	△	(*p* = 0.0008)
Away	50 cm (1.28)	9			10	△	(*p* = 0.0414)	1	▼	(*p* = 0.0081)
	55 cm (1.40)	9			6			5		
	60 cm (1.53)	7			1	▼	(*p* = 0.0290)	12	△	(*p* = 0.0379)
	65 cm (1.66)	4			1	▼	(*p* = 0.0353)	14	△	(*p* = 0.0064)
Empty-frame	50 cm (1.28)	8			8			4		
	55 cm (1.40)	7			5			8		
	60 cm (1.53)	5			3			12	△	(*p* = 0.0379)
	65 cm (1.66)	2	▼	(*p* = 0.0290)	1	▼	(*p* = 0.0290)	17	△	(*p* = 0.0002)

*“△” in this table flags the value as being significantly larger than the others in the same line (*p* < 0.05) and “**▼**” flags the value as being significantly smaller than the others in the same line (*p* < 0.05).*

A chi-squared test was conducted to determine if the preferred direction of shoulder rotation for the aperture of 65 cm (π = 1.66) or less differs depending on the configuration conditions. A significant interaction [χ*^2^*(54) = 202.880, *p* < 0.001, *Cramer*’*s V* = 0.426] was identified (Figure 7). Table 3 presents the results of the residual analysis (*p* < 0.05). A difference was noted in the preferred directions of shoulder rotation in the leftward and rightward conditions.

### Distance to the Aperture at Shoulder Rotation Onset

**FIGURE 8:**
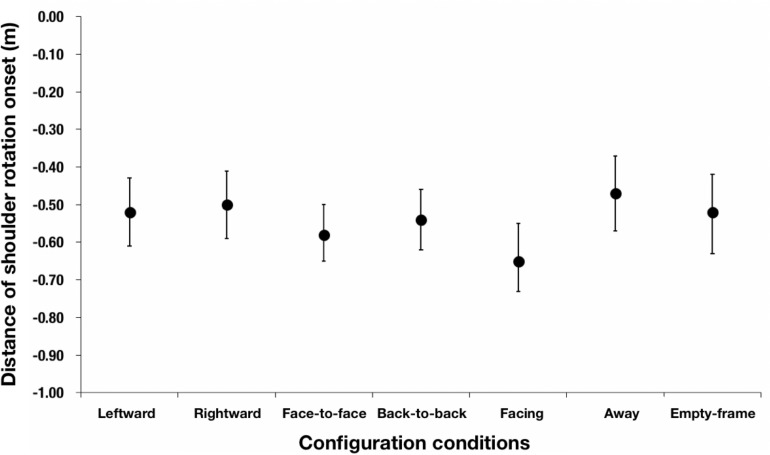
Mean distance to the aperture of shoulder rotation onset obtained using bootstrap mean and 95% bootstrap CI with the BCa method. The error bars indicate 95% bootstrap CI. The distance to the aperture at shoulder rotation onset in the facing condition was larger than that in the away condition because there was a small overlap in the 95% bootstrap CI with BCa method of the two configurations.

We compared the distance to the aperture at shoulder rotation onset by configuration conditions, using the data for the aperture of 65 cm (π = 1.66) or less; this is because shoulder rotation seldom occurred for the aperture of 70 cm (π = 1.79) or higher. It was difficult to conduct an analysis of variance because there were many missing values when the criterion of significant shoulder rotation was applied. Thus, a bootstrapping analysis was carried out for the present purpose. Table 4 presents the bootstrap mean and 95% bootstrap CI obtained with the BCa (Bias Corrected, accelerated) method (repetitions = 10000) (Efron, 1987). The results show that the distance to the aperture at shoulder rotation onset in the facing condition was larger than that in the away condition (Figure 8). There was a significant difference in the BCa bootstrap paired-samples test results between the onset distance of shoulder rotation in the facing condition and that in the away condition [*p* < 0.001, 95% CI on the mean of the differences [0.07, 0.29], repetitions = 10000], which suggests that the participants started rotating their shoulder earlier in the facing condition than in the away condition, implying that they took into consideration the anisotropic personal space.

**TABLE 4:** Mean distance to the aperture of shoulder rotation onset [bootstrap mean and 95% bootstrap CI with BCa method].

**Configuration conditions**	**Mean distance to the aperture of shoulder rotation onset (m)**
Leftward	−0.52 [−0.61, −0.43]
Rightward	−0.50 [−0.59, −0.41]
Face-to-face	−0.58 [−0.65, −0.50]
Back-to-back	−0.54 [−0.62, −0.46]
Facing	−0.65 [−0.73, −0.55]
Away	−0.47 [−0.57, −0.37]
Empty-frame	−0.52 [−0.63, −0.42]

*Means are bootstrap means and values in square bracket are 95% bootstrap CI. The number of repetitions for bootstrap was 10000.*

### Deviation of the Medial-Lateral Position (MLP) From the Center Line Between the Box-Shaped Frames

Table 5 presents the mean maximal deviation of the MLP from the center line between the box-shaped frames and 95% CI in each condition and each aperture width. A positive value of the mean maximal deviation of the MLP from the center line indicates that the participant tended to diverge to the left side, and a negative sign indicates that the participant tended to diverge to the right. The results show that all participants tended to diverge to the right side in all configuration conditions and all aperture width conditions in this experimental setting.

**TABLE 5:** Mean maximal deviation of the Medial-Lateral Position (MLP) from the center line between the box-shaped frames (m).

**Configuration conditions**	**Aperture width**						
	**50 cm**	**55 cm**	**60 cm**	**65 cm**	**70 cm**	**75 cm**	**80 cm**
Rightward	−0.0283 [−0.037, −0.002]	−0.0244 [−0.029, −0.020]	−0.0243 [−0.030, −0.019]	−0.0304 [−0.038, −0.023]	−0.0311 [−0.037, −0.025]	−0.0250 [−0.034, −0.016]	−0.0454 [−0.057, −0.034]
Facing	−0.0185 [−0.028, −0.009]	−0.0163 [−0.024, −0.009]	−0.0188 [−0.025, −0.012]	−0.0212 [−0.028, −0.014]	−0.0201 [−0.026, −0.014]	−0.0208 [−0.030, −0.012]	−0.0275 [−0.033, −0.022]
Back-to back	−0.0278 [−0.036, −0.019]	−0.0274 [−0.033, −0.022]	−0.0186 [−0.025, −0.013]	−0.0166 [−0.021, −0.012]	−0.0155 [−0.024, −0.007]	−0.0087 [−0.015, −0.003]	−0.0274 [−0.039, −0.016]
Away	−0.0243 [−0.032, −0.016]	−0.0230 [−0.031, −0.015]	−0.0179 [−0.023, −0.013]	−0.0125 [−0.016, −0.009]	−0.0197 [−0.028, −0.012]	−0.0181 [−0.028, −0.009]	−0.0223 [−0.031, −0.013]
Empty-frame	−0.0242 [−0.030, −0.018]	−0.0229 [−0.026, −0.020]	−0.0238 [−0.027, −0.020]	−0.0120 [−0.016, −0.008]	−0.0266 [−0.034, −0.019]	−0.0189 [−0.026, −0.013]	−0.0201 [−0.030, −0.011]
Face-to-face	−0.0221 [−0.030, −0.014]	−0.0225 [−0.029, −0.016]	−0.0163 [−0.023, −0.010]	−0.0161 [−0.021, −0.011]	−0.0193 [−0.029, −0.010]	−0.0182 [−0.026, −0.011]	−0.0166 [−0.021, −0.012]
Leftward	−0.0194 [−0.034, −0.005]	−0.0175 [−0.023, −0.012]	−0.0117 [−0.017, −0.007]	−0.0057 [−0.013, 0.001]	−0.0008 [−0.009, 0.007]	−0.0065 [−0.015, 0.002]	−0.0019 [−0.008, 0.004]

*Values in brackets indicate 95% confidence intervals.*

**FIGURE 9:**
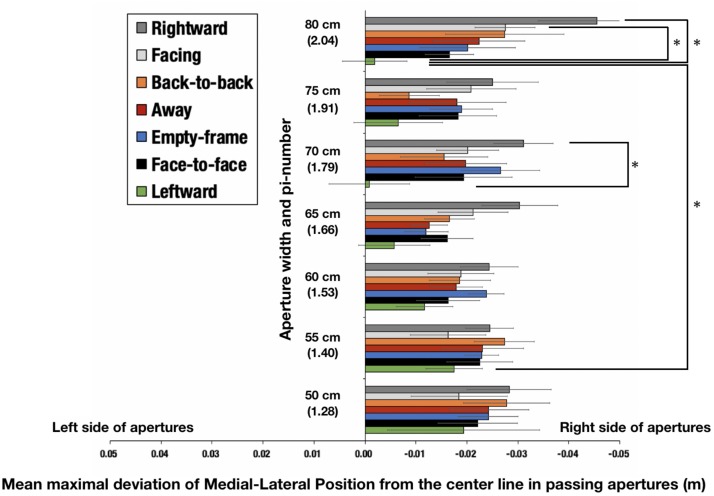
Mean maximal deviation of the Medial-Lateral Position (MLP) from the center line in passing apertures. The parenthetical number indicates pi-number, i.e., aperture width divided by the mean shoulder width of the participants. The error bars indicate 95% CI. For the aperture width of 70 cm (π = 1.79), the mean maximal deviation of the MLP from the center line in passing apertures in the leftward condition was more divergent toward the left than it was in the rightward condition (*adjusted p* = 0.0287). For the aperture width of 80 cm (π = 2.04), the mean maximal deviation of the MLP from the center line magnitude of divergence in passing apertures in the leftward condition was more divergent toward the left than it was in the rightward (*adjusted p* = 0.0048) and facing conditions (*adjusted p* = 0.0061). The mean maximal deviation of the MLP from the center line magnitude of divergence in passing apertures in the leftward condition of 80 cm (π = 2.04) was more divergent toward the left than that in the leftward condition of 55 cm (π = 1.40) (*adjusted p* = 0.0374).

A two-way repeated measures analysis of variance (7 configurations × 7 aperture widths) was conducted using the average values. Significant interactions [*F*(36,324) = 1.98, *p* = 0.0038, $\eta_{G}^{2}$ = 0.0483, *power* = 0.6807] were identified (Figure 9). Significant main effects of configurations [*F*(6,54) = 7.7781, *p* < 0.001, $\eta_{G}^{2}$ = 0.0748, *power* = 1.0000] were also identified. There were no significant main effects of aperture-widths [*F*(6,54) = 1.1630, *p* = 0.3397, $\eta_{G}^{2}$ = 0.0190, *power* = 0.6493]. Application of the Holm–Bonferroni method revealed that, for the aperture width of 70 cm (π = 1.79), the mean maximal deviation of the MLP from the center line in passing apertures in the leftward condition was more divergent toward the left than it was in the rightward condition (*adjusted p* = 0.0287). Moreover, for the aperture width of 80 cm (π = 2.04), the mean maximal deviation of the MLP from the center line in passing apertures in the leftward condition was more divergent toward the left than that it was in the rightward (*adjusted p* = 0.0048) and facing conditions (*adjusted p* = 0.0061). Furthermore, the mean maximal deviation of the MLP from the center line in passing apertures in the leftward condition of 80 cm (π = 2.04) was more divergent toward the left than it was in the leftward condition of 55 cm (π = 1.40) (*adjusted p* = 0.0374).

### Approaching Velocity

In this analysis, the average of the two values of approaching velocity was used as the representative value. Table 6 shows the average values of approaching velocity and 95% CI in each condition and each aperture width. A two-way repeated measures analysis of variance (7 configurations × 7 aperture widths) was conducted using the average values of approaching velocity. There were no significant main effects of configurations [*F*(6,54) = 1.5927, *p* = 0.1671, $\eta_{G}^{2}$ = 0.0046, *power* = 0.9629] and aperture-widths [*F*(6,54) = 0.4540, *p* = 0.8390, $\eta_{G}^{2}$ = 0.0008, *power* = 0.2290]. A trend for interaction [*F*(36,324) = 1.3920, *p* = 0.0729, $\eta_{G}^{2}$ = 0.0043, *power* = 0.5032] was identified. Results of the Holm–Bonferroni test revealed that, for the aperture width of 65 cm (π = 1.66), approaching velocity in the facing condition was higher than that in the leftward condition (*adjusted p* = 0.0305); however, the power was low.

**TABLE 6:** Mean approaching velocity (m/s).

**Configuration conditions**	**Aperture width**						
	**50 cm**	**55 cm**	**60 cm**	**65 cm**	**70 cm**	**75 cm**	**80 cm**
Leftward	1.21 [1.19,1.23]	1.20 [1.18,1.22]	1.20 [1.19,1.21]	1.19 [1.18,1.21]	1.21 [1.20,1.22]	1.22 [1.20,1.24]	1.23 [1.21,1.25]
Rightward	1.23 [1.21,1.25]	1.24 [1.22,1.26]	1.24 [1.22,1.26]	1.23 [1.20,1.25]	1.23 [1.21,1.25]	1.22 [1.21,1.24]	1.19 [1.16,2.22]
Face-to-face	1.21 [1.17,1.25]	1.21 [1.19,1.23]	1.21 [1.19,1.22]	1.22 [1.20,1.23]	1.23 [1.22,1.23]	1.21 [1.20,1.23]	1.24 [1.22,1.25]
Back-to-back	1.22 [1.20,1.25]	1.22 [1.20,1.24]	1.23 [1.20,1.25]	1.23 [1.22,1.25]	1.24 [1.22,1.26]	1.23 [1.22,1.25]	1.23 [1.21,1.25]
Facing	1.23 [1.20,1.25]	1.22 [1.20,1.24]	1.24 [1.22,1.27]	1.24 [1.22,1.27]	1.23 [1.21,1.25]	1.24 [1.22,1.26]	1.25 [1.23,1.28]
Away	1.20 [1.17,1.23]	1.21 [1.18,1.23]	1.20 [1.18,1.21]	1.20 [1.18,1.21]	1.21 [1.19,1.23]	1.22 [1.20,1.23]	1.22 [1.20,1.24]
Empty-frame	1.21 [1.20,1.23]	1.22 [1.21,1.24]	1.23 [1.22,1.24]	1.22 [1.20,1.23]	1.21 [1.19,1.23]	1.23 [1.20,1.25]	1.24 [1.21,1.26]

*Values in brackets indicate 95% confidence intervals.*

### Passing Velocity

In this analysis, the average of the two values of passing velocity was taken as the representative value. Table 7 lists the average values of passing velocity and 95% CI in each condition and each aperture width. A two-way repeated measures analysis of variance (7 configurations × 7 aperture widths) was conducted using the average values of passing velocity. There were no significant main effects of configurations [*F*(6,54) = 1.0458, *p* = 0.4064, $\eta_{G}^{2}$ = 0.0061, *power* = 0.8440] and aperture-widths [*F*(6,54) = 0.1639, *p* = 0.9852, $\eta_{G}^{2}$ = 0.0023, *power* = 0.0946]. No significant interactions [*F*(36,324) = 0.8152, *p* = 0.7679, $\eta_{G}^{2}$ = 0.0100, *power* = 0.2962] were identified.

**TABLE 7:** Mean passing velocity (m/s).

**Configuration conditions**	**Aperture width**						
	**50 cm**	**55 cm**	**60 cm**	**65 cm**	**70 cm**	**75 cm**	**80 cm**
Leftward	1.19 [1.11,1.26]	1.19 [1.16,1.21]	1.20 [1.17,1.24]	1.18 [1.16,1.20]	1.19 [1.15,1.22]	1.19 [1.15,1.22]	1.20 [1.16,1.24]
Rightward	1.21 [1.14,1.27]	1.22 [1.18,1.26]	1.22 [1.19,1.26]	1.19 [1.15,1.23]	1.21 [1.18,1.24]	1.21 [1.18,1.23]	1.18 [1.13,1.22]
Face-to-face	1.20 [1.14,1.27]	1.20 [1.16,1.24]	1.19 [1.16,1.23]	1.21 [1.18,1.23]	1.21 [1.20,1.23]	1.22 [1.19,1.24]	1.23 [1.20,1.26]
Back-to-back	1.20 [1.12,1.29]	1.18 [1.15,1.22]	1.20 [1.18,1.24]	1.21 [1.18,1.24]	1.23 [1.20,1.25]	1.22 [1.20,1.24]	1.22 [1.19,1.25]
Facing	1.22 [1.15,1.29]	1.20 [1.17,1.23]	1.21 [1.18,1.23]	1.21 [1.17,1.23]	1.19 [1.16,1.22]	1.21 [1.17,1.24]	1.20 [1.17,1.24]
Away	1.20 [1.14,1.26]	1.13 [1.10,1.15]	1.19 [1.16,1.21]	1.18 [1.16,1.20]	1.19 [1.17,1.21]	1.19 [1.17,1.21]	1.18 [1.15,1.21]
Empty-frame	1.19 [1.15,1.23]	1.17 [1.16,1.19]	1.19 [1.16,1.22]	1.23 [1.20,1.26]	1.19 [1.16,1.23]	1.21 [1.17,1.25]	1.19 [1.16,1.23]

*Values in brackets indicate 95% confidence intervals.*

### Critical Pi-Number of Maximum Shoulder Rotation

The critical aperture width at which the maximum shoulder rotation changes was determined, with reference to Warren and Whang (1987) and Hackney et al. (2015), by taking the aperture width immediately preceding the points for which statistically significant differences were not confirmed. The critical pi-number is the critical aperture width divided by the average shoulder width of the participants. In this study, the critical pi-number in the face-to-face condition was 1.66, that in the back-to-back condition 1.53, that in the facing condition 1.66, that in the away condition 1.40, that in the leftward condition 1.53, that in the rightward condition 1.53, and that in the empty-frame condition was 1.40. These pi-numbers were the critical aperture widths divided by the average shoulder width of the participants; thus, the difference between the conditions was the same as the results of the shoulder rotation angle section. Therefore, the shoulder angle in the face-to-face condition was significantly larger than that in the empty-frame condition (*adjusted p* = 0.0492).

## Discussion

This study investigated how an individual controlled his behavior while passing through an aperture, according to the configuration of the two humans constituting the aperture, with the shape of the aperture being controlled to be the same across all the conditions tested. This manipulation allowed us to infer, without attributing the results to different shapes of an aperture in different configuration conditions, how people perceived and treated the anisotropic personal space of two humans in the considered task. The following section summarizes and discusses the results obtained.

First, we found that differences in the mutual orientation of confederates affects walkers’ shoulder rotation angle (see section Shoulder Rotation and Its Angle). Particularly, the magnitude of the shoulder rotation angle in the face-to-face condition was larger than that in the empty-frame condition (Figure 6). This suggests that the participants observed, wittingly or unwittingly, the anisotropic personal space in front of the confederates’ body that overlaps with the aperture in the face-to-face condition, making them feel it more difficult to pass through the aperture in this condition as if it were a form of “intrusion” than in any of the other conditions. Conversely, it is easier to pass through the aperture in the other conditions (including the empty-frame condition) than in the face-to-face condition, because in the former conditions there is less, or an absolute lack of, personal space on which to intrude.

Second, the preferred direction of shoulder rotation differed depending on the configuration of the confederates. In particular, in the leftward condition, the counterclockwise rotation was preferred when the aperture width was less than 65 cm (π = 1.66). Similarly, in the rightward condition, the clockwise rotation was preferred when the aperture width was less than 65 cm (π = 1.66). This implies that although the shape and the width of the apertures were controlled to be the same using the box frame, the participants’ action changed as a function of the configuration of confederates. Moreover, constrained by the frames, the humans in the box frame could not move their body, and were instructed not to move their head. The preferred direction of shoulder rotation may have changed not only because of personal space but also because of visibility of the human confederates’ faces. That is, the participants possibly wished to, wittingly or unwittingly, avoid approaching too close to the confederate facing inward to the aperture, which could be accomplished if the participant rotated their shoulder counterclockwise or clockwise. The significance of the confederates’ faces as a factor of aperture passability judgment will be investigated in future work.

Third, the distance to the aperture at shoulder rotation onset differed depending on the configuration of the confederates. In particular, as shown in Figure 8, shoulder rotation occurred farther away from the aperture in the facing condition than in the away condition when the aperture width was 65 cm (π = 1.66) or less. This suggests that the participants perceived the confederates’ anisotropic personal space and started rotating their shoulder earlier to avoid it.

Fourth, We found that configuration of the confederates, in other words anisotropic personal space influences the deviation of the MLP from the center line while passing through the aperture [see section Deviation of the Medial-Lateral Position (MLP) From the Center Line Between the Box Shaped Frames]. The deviation of MLP from the center line while passing through the aperture in the leftward and rightward conditions, among other things, were influenced by the default MLP (which was slightly off the center, to the right) toward the left and right, respectively, when the aperture was relatively wide. This tendency was not clearly demonstrated statistically in the relatively narrow aperture condition because, presumably, there was less or no room to adjust the trajectory in these conditions.

Fifth, given that there was no main effect of the confederates’ configuration on the velocity of approaching or passing through an aperture across different conditions, it is plausible that individuals adjust in accordance with different conditions by changing either the direction of shoulder rotation or the adopted route, in order to pass through an aperture. Furthermore, the participants tended to prefer one of those strategies more than the other. When the aperture width was more than 70 cm (π = 1.79), the participants appeared to prefer using the “trajectory” strategy although the “shoulder rotation” strategy was similarly available, whereas for an aperture width less than 65 cm (π = 1.66), they tended to employ the “shoulder rotation” strategy because, perhaps, the limited space does not allow the implementation of the “trajectory” strategy.

Sixth, the critical pi-number of maximum shoulder rotation differed depending on the configuration of the confederates. The critical pi-number in the facing and face-to-face conditions (π = 1.66) in this study is comparable to that reported for the human obstacle condition in Hackney et al.’s (2015) study (π = 1.7). Moreover, the critical pi-number in the empty-frame condition (π = 1.40) in this study is approximately the same as that reported by Warren and Whang (1987) (π = 1.30) and Hackney et al. (2015) (π = 1.3), wherein the participants passed between two obstacles.

As mentioned in the Introduction of this paper, Gibson and Crooks (1938) discussed the impact of the presence of others on one’s driving action. When driving a car, we can drive safely without colliding with obstacles. We can perceive the field of possible paths such that our vehicle is unimpeded—this is called the field of safe travel (Gibson and Crooks, 1938). The field of safe travel has an anisotropic structure in that it tends to be longer toward the front of the vehicle than it does toward the sides and at the rear. This is because a vehicle that is not in motion is more likely to start moving forward than backward or sideways. At an intersection where there is more than one vehicle, the fields of safe travel may overlap with each other. To a certain driver, the field of safe travel of another’s vehicle manifests itself as having negative valence (Gibson and Crooks, 1938), because that is the path that must not be taken to avoid a collision. In such a situation, one’s field of safe travel is reorganized, or deformed, by integrating the field of safe travel of the other vehicle (with negative valence). The drivers need to perceive the reorganized field of safe travel to keep driving safely. This idea may help explain the findings of the present study, which suggest that the presence of two standing humans’ anisotropic personal space affects the way in which an individual passes between them.

In an attempt to explain steering, obstacle avoidance, or route selection, Fajen and Warren (2003) and Fajen et al. (2003) proposed the Behavioral Dynamics Model, which describes the steering behavior of an agent. The model determines how an agent’s egocentric location of a goal contributes to the angular acceleration of steering, and it is assumed to be a function of the goal angle, goal distance, obstacle angle, and obstacle distance (Fajen and Warren, 2003; Fajen et al., 2003). This model precisely captures visually guided walking in humans under certain ideal conditions, but it treats the agent, goal, and obstacles as mathematical points without any extension (i.e., unlike corporeal objects) and/or as having no structured (anisotropic or otherwise) field. Considering the present findings regarding the impact of anisotropic personal space on an agent’s aperture passing behavior and the idea of a reorganized field of safe travel discussed above, the Behavioral Dynamics Model can be extended to more realistic cases wherein an agents’ behavior is subject to the field of safe travel and the personal space of other agents (vehicles, people, etc.).

## Future Work

In analyzing the data, the gaze and/or the direction of the face of the confederates emerged as a possible factor relevant to how one passes between two humans. Different results could have been obtained by controlling the gaze condition (e.g., by covering the confederates’ face with a face-size screen), if, as indicated previously, the preferred direction of shoulder rotation was affected by the face or gaze of two standing humans. This would elucidate another dimension of the influence of social factors on the passing behavior of an aperture composed of two humans. We will examine this experimentally in future work.

## Conclusion

This study investigated the manner in which individuals pass between two humans standing in various configurations. The results suggest that when the aperture width is more than 70 cm (π = 1.79), the participants tend to prefer to use the “trajectory” strategy although the “shoulder rotation” strategy is similarly available. In contrast, the “shoulder rotation” strategy is preferred when the aperture width is less than 65 cm (π = 1.66), owing to the space not being sufficiently large to adopt the “trajectory” strategy. This study can be considered as an attempt to describe quantitatively how individuals select one action from several possible strategies available in a given environment. In particular, the results highlight that individuals adjust their strategies pertaining to passing between two humans, as per not only the physical constraints (e.g., ratio of aperture width to shoulder width), but also the socio-cultural constraints (e.g., anisotropic shape of personal space) of what constitutes the aperture.

## Data Availability Statement

All datasets generated for this study are included in the article/supplementary material.

## Ethics Statement

The studies involving human participants were reviewed and approved by the Academic Research Ethical Review Committee at Waseda University. This study was carried out in accordance with the recommendations of the Academic Research Ethical Review Committee at Waseda University with written informed consent from all subjects. All subjects gave written informed consent in accordance with the Declaration of Helsinki. The protocol was approved by the Academic Research Ethical Review Committee at Waseda University. The patients/participants provided their written informed consent to participate in this study.

## Author Contributions

TT, RM, HM, and NF conceived and designed the study. TT conducted the experiment and wrote the manuscript. TT and HM analyzed the data. RM, NF, and HM contributed to the improvement of the manuscript.

## Conflict of Interest

The authors declare that the research was conducted in the absence of any commercial or financial relationships that could be construed as a potential conflict of interest.

## Footnotes

1 The participants and the confederates were all male. This is because there are gender differences in personal space (Aono, 2003). This factor will be examined in future work.

2 The aperture widths could be represented in pi-number, or π for short, (aperture width/participants’ average shoulder width: 39.2 cm); 1.28 (aperture width: 50 cm/participants’ average shoulder width: 39.2 cm), 1.40 (55 cm/39.2 cm), 1.53 (60 cm/39.2 cm), 1.66 (65 cm/39.2 cm), 1.79 (70 cm/39.2 cm), 1.91 (75 cm/39.2 cm), 2.04 (80 cm/39.2 cm).
